# Evaluating refugia in recent human evolution in Africa

**DOI:** 10.1098/rstb.2020.0485

**Published:** 2022-04-25

**Authors:** James Blinkhorn, Lucy Timbrell, Matt Grove, Eleanor M. L. Scerri

**Affiliations:** ^1^ Pan-African Evolution Research Group, Max Planck Institute for the Science of Human History, Jena, Germany; ^2^ Centre for Quaternary Research, Department of Geography, Royal Holloway, University of London, Egham, UK; ^3^ Department of Archaeology, Classics and Egyptology, University of Liverpool, Liverpool, UK; ^4^ Department of Classics and Archaeology, University of Malta, Msida, Malta; ^5^ Institute of Prehistoric Archaeology, University of Cologne, Cologne, Germany

**Keywords:** refugia, Late Pleistocene, Africa, *Homo sapiens*, palaeoenvironmental change

## Abstract

*Homo sapiens* have adapted to an incredible diversity of habitats around the globe. This capacity to adapt to different landscapes is clearly expressed within Africa, with Late Pleistocene *Homo sapiens* populations occupying savannahs, woodlands, coastlines and mountainous terrain. As the only area of the world where *Homo sapiens* have clearly persisted through multiple glacial-interglacial cycles, Africa is the only continent where classic refugia models can be formulated and tested to examine and describe changing patterns of past distributions and human phylogeographies. The potential role of refugia has frequently been acknowledged in the Late Pleistocene palaeoanthropological literature, yet explicit identification of potential refugia has been limited by the patchy nature of palaeoenvironmental and archaeological records, and the low temporal resolution of climate or ecological models. Here, we apply potential climatic thresholds on human habitation, rooted in ethnographic studies, in combination with high-resolution model datasets for precipitation and biome distributions to identify persistent refugia spanning the Late Pleistocene (130–10 ka). We present two alternate models suggesting that between 27% and 66% of Africa may have provided refugia to Late Pleistocene human populations, and examine variability in precipitation, biome and ecotone distributions within these refugial zones.

This article is part of the theme issue ‘Tropical forests in the deep human past’.

## 1. Introduction

Today, humans occupy an incredible diversity of habitats. With the exception of Antarctica, *Homo sapiens* had colonized all continents by the start of the Holocene, expanding into new and unfamiliar landscapes, such as the remote island chains of the Pacific Ocean, within a relatively short time frame. Examining and explaining this capacity to expand into new habitats is central to understanding why we are now the only extant human population, in contrast with the hominin landscape at the start of the Late Pleistocene. While the clearest expression of this ecological plasticity may be seen in our expansions ‘Out of Africa’, the foundations for this were laid in our adaptation to and engagement with African landscapes. Considerable ecological diversity can be observed across Africa and examining how human populations may have interacted with this diversity presents a critical step to understanding what promoted our potential for extreme ecological plasticity. Historically, significant focus has been placed on eastern Africa owing to the distribution of early fossil specimens of *Homo sapiens* alongside its geographical placement as both a key region to examine patterns of ecological adaptation and a potential centre for dispersals into Eurasia [1,2]. While some more recent studies have identified single geographically circumscribed regions as putative centres for the origins of our species [3], these appear untenable [4]. Instead, there is a growing appreciation of the role played by interaction between sub-structured populations across the continent in human origins and the engagement of local populations with different ecologies [5,6]. Identifying the ecological contexts sustaining human habitation within Africa is central to exploring how and when expansions into new landscapes may have occurred, and what promoted them.

The dynamics of population expansion and contraction in response to broad-scale climatic changes, as well as subsequent climatic impacts on population diversity, are frequently examined through the lens of refugia models. The term ‘refugia’ has been widely adopted by palaeoanthropologists [7] and is employed to describe those landscapes and ecological contexts supporting enduring human presence in the face of global climatic change. It is, however, rare that either the ecological parameters for such refugia are made explicit, or that models for the potential impact of refugia are clearly contrasted with other hypothetical explanations for changes in population distributions through time. Here, we review how human refugia have been identified in the Late Pleistocene record of Africa and, through the application of modelled datasets spanning 130–10 ka, aim to identify potential climatic and ecological parameters for such refugia.

## 2. The role of refugia in African palaeoanthropology

The terms ‘*refugia*’ and ‘*refugium*’ were first used by ecologists in the mid-twentieth century examining the contraction of plant ranges during the Last Glacial Maximum (LGM) in North America [8] and have since gone on to be widely applied in the study of fluctuating population distributions in a broad variety of environments [9–11], including multiple discrete studies on recent human evolution [12–15]. Though species vary in their exact responses to habitat perturbation, all mammals are habitat-specific [16] and thus are affected by environmental change, making the study of refugia vital for understanding animal population dynamics.

The core concept of refugia is that a species', or population's, potential geographical range can become reduced under conditions of climatic stress, such as the LGM, which reduces the area of suitable habitat. A refugium is an area in which a given habitat is present throughout the period of climatic stress, enabling population persistence. Outside refugia, a population may respond to climatic stress in several ways. These include tracking the changing distribution of their preferred habitat, adapting to new climatic conditions or undergoing repeated vicariance and eventually becoming extinct [16,17]. At the simplest level, then, the concept of refugia links together the modulation of population distributions by wider patterns of environmental change and contrasts the two extremes of the spectra of distribution and environmental conditions—the refugium in which a population persists during the most extreme climatic stress, and the wider range it retreats from and returns to as this stress is relieved. In practice, the fragmentation of a population's range may produce multiple, disconnected habitable areas that vary both in size (macro- versus micro-refugia) and proximity to one another, leading to the formation of allopatric populations. Such population vicariance increases variation and divergence within a species without speciation through both natural selection and genetic drift [16–19]. Referred to as ‘evolutionary geography’ by Lahr & Foley [18], this role of spatio-temporal biogeographical factors in driving micro-evolutionary processes is supported by the evolutionary history of Africa fauna (e.g. [20,21]). Studies of African fauna demonstrate how the fragmentation and coalescence of whole ecosystems during alternate phases of climatic stress and amelioration influences the extent and nature of population range expansion and contraction, and can ultimately shape the phylogenetic structure of population diversity within a species [16–18]. Within ecological studies, such refugia models provide a key tool in studies of taxonomy, phylogenetic speciation and changing population distributions that can be contrasted against a null model of stochastic variability and alternate explanatory models for variability, such as isolation by distance.

A number of factors have confounded the straightforward deployment of refugia models to examine human biogeography, particularly in Africa. Refugia models typically focus on the climatic extremes of glacial-interglacial cycles to characterize a population's maximum and minimum range (e.g. [11]). The use of glacial-interglacial cycles as the time frame for classical refugia models restricts wider examination of refugia for *Homo sapiens* to Africa, as the only place where the populations of this species clearly persisted throughout the Late Pleistocene. However, it is worthwhile noting that 100 thousand year glacial-interglacial cycles may not best characterize the tempo and patterning of the extremes of palaeoclimatic change across sub-Saharan Africa [22]. Persistent occupations, as well as conspicuous periods without clear evidence for occupation, have been identified by archaeologists to help identify putative refugial zones within Africa. For instance, in northeast Africa, Prendergast *et al*. [23] suggest increases of occupation density at Haua Fteah during Marine Isotope Stage (MIS) 2 resulted from a more widespread population seeking refugia. Similarly, Marean [24] suggests that intensive occupation of the southern African Cape is a result of a population crash in the arid interior. Bushozi *et al*. [25] suggest that the long continuity of occupation at Mumba, eastern Africa, indicates its location within a refugium; conversely, Campmas [26] and Leplongeon *et al*. [27] question whether patterns of discontinuity of occupation in the Maghreb and Horn of Africa, respectively, may be explained by retreat to refugia located elsewhere. Identifying refugia demands clear characterization of spatial and temporal patterns of population presence and absence; both become more problematic to evaluate with increasing time depth and are filtered through practices of archaeology and histories of research.

Within tropical and sub-tropical zones, the accessibility of water is widely considered to be a key factor limiting human ranges, with variability in precipitation having a dominant role in regulating low-latitude habitability as opposed to temperature. A range of African landscapes and landscape settings have been proposed to have offered refugia to recent human populations based on the continued availability of water resources, and similarly landscapes prone to extreme aridification are contra-indicated for long-term habitability. For instance, fluvial systems, such as the Zambezi [28] and Omo river [29], have been proposed to have provided important refugia for early *Homo sapiens* populations. Leplongeon [30] argues that the Nile Valley was more likely to have been a refugium than a corridor during MIS 2, though modelling work by Beyer *et al*. [31] suggested that dispersals out of the continent would have been impossible without the presence of the Nile. The Rift Valley of eastern Africa is known for its major lake systems that would have provided stable sources of water during dry phases, supporting human populations in the long term [32,33]. These Rift Valley water sources probably acted both as catalysts and barriers to human interaction [34], as they do ethnographically [35]. High-altitude landscapes have been highlighted as potential refugia owing to their enhanced humidity in contrast with surrounding lowlands, such as in the Ethiopian Rift [36,37] or the Lesotho Highlands [38,39]. Similarly, enhanced precipitation experienced in proximity to the coast has been indicated as a feature that may have supported enduring occupation of sites in eastern and southern Africa (e.g. [40,41]). Cuthbert *et al*. [42] highlight the potential importance of persistent groundwater-fed hydro-refugia to buffer climatic flux in eastern Africa, enabling occupation of landscapes that may otherwise be considered too dry based solely on patterns of precipitation—a potential example of crypto-refugia. Beyond identification of geographical features that may provide access to water resources, modelled climate conditions can help to parameterize conditions that were habitable to human populations. For instance, Blinkhorn & Grove's [43] examinations of LGM and MIS5e datasets (as proxies for extremes of aridity and humidity, comparable to standard refugia models) identify Middle Stone Age (MSA) occupations in eastern Africa during interglacial conditions as being more numerous and occurring in landscapes with a broader range of precipitation values (145–1350 mm yr^−1^) compared to the rarer occupations in glacial phases (675–880 mm yr^−1^).

That the availability of water has repeatedly been identified as a key constraint on the distribution of human populations within Late Pleistocene Africa is perhaps unsurprising. This may, in part, reflect the scarcity of wider data on which to develop more complex models of resource use and adaptation to environmental conditions. What is perhaps surprising, however, is the breadth of landscapes that, through the persistent availability of water, have been proposed as potential refugia for human populations, with only those landscapes prone to severe aridity notably absent. A range of studies suggest that it is the accessibility of woodlands, which also require sufficient precipitation to flourish, that played an important role in supporting the long-term continuity of African human populations. Historically, tropical forests have often been conceived of as ‘green deserts’, lacking suitable resource structures to sustain Pleistocene foraging populations and only becoming habitable with the advent of agriculture [44]. This conflicts with varying suggestions that archaeological taxa such as the Sangoan and Lupemban may have been intimately tied to the occupation of tropical forests, or perhaps their margins. However, our understanding of the diversity of these phenomena and their chronology remains limited [45]. Basell [1] presents one of the most lucid applications of refugia models to the eastern African MSA record, examining modelled patterns of ecological change between peak glacial and interglacial conditions, and highlights the significance of access to woodlands during arid phases for the continuity of human populations. Recent research at Panga ya Saidi [40,46] supports this hypothesis, with enduring occupation of the site throughout the Late Pleistocene associated with the use of forest resources. Recent, significant advances to the Stone Age records of West Africa also highlight how history of research has played a significant role in shaping concepts of human refugia in Africa which potentially overlook considerable past diversity in certain underexplored regions [47]. Roberts & Stewart [48] survey the breadth of ecologies inhabited by later Pleistocene African hunter–gatherers, highlighting the potential importance of being able to access different resource bases rather than focussing on any single habitat. For example, the heterogeneity of landscapes and ecologies present in the Central Rift Valley, where the intersection of multiple lake basins set in varying topographic contexts promotes the continuity of stable but diverse resources [49,50]. Ecotones, denoting the intersection of alternate ecologies, may be of particular importance for understanding regions of enduring habitability for Late Pleistocene human populations.

## 3. Exploring potential human refugia in Late Pleistocene African landscapes

Refugia have been broadly implicated in African palaeoanthropology, but, as the foregoing review indicates, the application of refugia models to examine Late Pleistocene human populations on the continent is not straightforward. The preceding section has identified tangible features that have been proposed as significant to support enduring occupation by later Pleistocene populations in Africa. First, humidity is widely acknowledged as a key parameter to understand human distributions. Second, accessibility of woodland habitats has repeatedly been suggested to have been vital for supporting persistent occupations by human populations, placing focus on the presence and distribution of ecotones. Here, we examine changing patterns of precipitation and biome distribution throughout the Late Pleistocene (130–10 ka), based on modelled mean annual precipitation (BIO12) and biome datasets presented by Krapp *et al*. [51,52]. These data enable spatially explicit examination of changes through time at a 0.5 × 0.5 degree and 1-thousand-year time slice resolution for paleoclimatic and habitat parameters that are directly meaningful for examining patterns of past human distributions, such as quantifying annual precipitation in mm. Regional proxy datasets may offer higher levels of chronological resolution and/or specificity regarding particular climatic or ecological conditions and are used to validate the model datasets employed here (see [51,52]). At present, however, no synthesis of proxy records provides comparable spatially explicit coverage of Africa throughout the Late Pleistocene, providing units of analysis that may be directly meaningful to examine patterns of past human distributions. Syntheses of empirical palaeoclimatic data across Africa (e.g. [53]) demonstrate substantial spatial asynchrony, with even geographically proximate core records demonstrating remarkable differences in their reconstructions over similar periods (cf. Lake Tana and Chew Bahir). Such syntheses are hampered by differences in the type and sophistication of age models, and by the use of multiple different proxies, many of which have nonlinear or incompletely characterized relationships with the palaeoenvironmental variables they represent. The quantitative description and analysis of refugia requires data that are continuous in both space and time; climate simulations currently provide the only such data available. Here, we focus on identifying regions for which precipitation persistently falls within parameters derived from ethnographic datasets throughout the Late Pleistocene as proposed refugia and explore variability in patterns of precipitation and biome variability through time within these core regions. We then test whether the proposed refugia correspond to patterns of habitability and continuity of occupation within eastern Africa as a case study.

### (a) Identifying precipitation refugia

Multiple previous studies highlight the flux of aridity and humidity as a key parameter influencing past population distributions for Late Pleistocene humans in Africa. In order to establish potential precipitation thresholds for Pleistocene forager population distributions, we employ a large ethnographic dataset (*n* = 235) [54], restricted solely to fully mobile, as opposed to partially sedentary, groups [55]. Despite the routine use of ethnographic data to parameterize past human distributions [31,56–58], we acknowledge that the ethnographic dataset used here may not reflect potential or preferred distributions among recorded populations, nor directly correspond to Pleistocene populations [59], and as a result we restrict our use of ethnographic data. In order to constrain the impact of such issues, we impose two standardly applied confidence intervals (CI) on the mean annual precipitation (BIO12) dataset spanning 68% and 95% of the range of conditions experienced by extant hunter–gatherers. This results in boundaries for forager population distributions of 248–1403 mm (CI 68%) and 127–3286 mm (CI 95%) total precipitation per annum. Blinkhorn & Grove [43] identified the range of mean annual precipitation values for eastern African MSA sites modelled under interglacial conditions to span 145–1350 mm, for which the upper threshold is broadly comparable with the upper 68% CI boundary, whereas the lower threshold is closer to the lower 95% CI boundary. Beyer *et al*. [60] impose a threshold of 90 mm for potential human habitation, highlighting that this also marks a key ecological threshold for sustaining grazing fauna, a value which falls between 95% and 99% CIs from the ethnographic data. Here, the 68% and 95% CIs, referred to hereafter as narrow refugia and broad refugia respectively, are used as tools to constrain the distribution of potential habitable landscapes during the Late Pleistocene as a step towards higher resolution diachronic analysis of human refugia.

**Figure 1.**
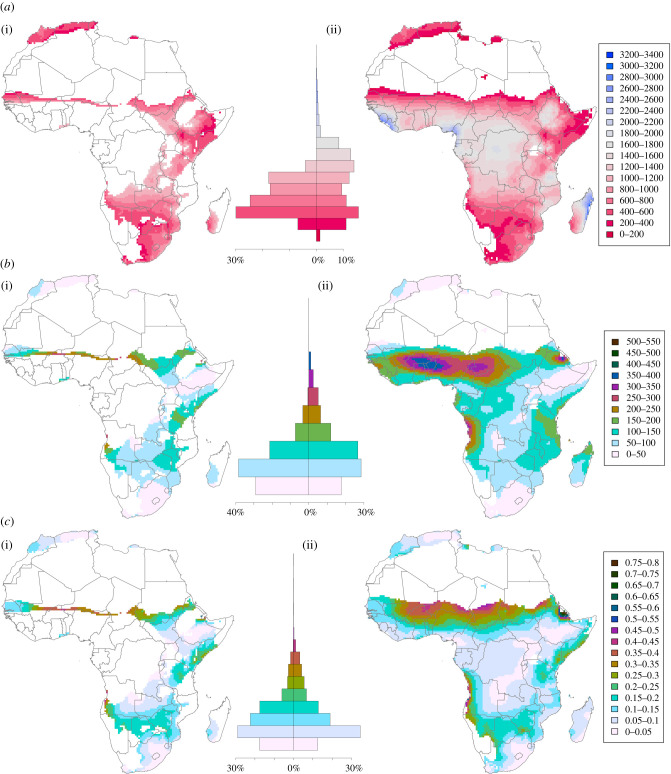
Maps of persistently habitable landscapes based on narrow refugia (i) and broad refugia (ii); illustrating (*a*) mean annual precipitation (mm), (*b*) s.d. of mean annual precipitation (mm), and (*c*) coefficient of variation of mean annual precipitation. Histograms (centre) display the percentage area of Africa covered within respective categories of mean annual precipitation, s.d. and coefficient of variation and within narrow refugia (centre-left) and broad refugia (centre-right).

We identified landscapes in Africa which persistently receive mean annual precipitation falling within each refugia throughout the Late Pleistocene (figure 1*a*), with habitable areas within the narrow refugia spanning 27.5% of the total area of Africa, increasing to 66.3% for the broad refugia. The Sahara, Kalahari and Namib deserts, as well as sparse locations within the Horn of Africa and on the coast of Sierra Leone and Liberia, never present persistently habitable conditions that fall within the broad refugia during the Late Pleistocene. Isolated persistently habitable landscapes are present on the northern African coast, yet most of sub-Saharan Africa is persistently habitable within the broad refugia. Within the narrow refugia, the largest, contiguous areas that appear persistently habitable throughout the Late Pleistocene are found in southeast and south-central Africa, with narrow points of connection with habitable regions spanning much of the Tanzanian and Kenyan Rift Valley and the regions surrounding the Ethiopian Rift/Highlands. A discontinuous latitudinal band of habitable landscapes spans the southern Sahel across to a more substantial habitable zone in the Senegal Valley. The Maghreb in northern Morocco and Algeria, as well as Cyrenaica in Libya, possess fragmented pockets of persistently habitable landscapes falling within the narrow refugia.

We examine patterns of precipitation within the two refugia with respect to the mean of Late Pleistocene precipitation, providing a rapid characterization of its variability (figure 1*a*). Within the narrow refugia, there is a left-skewed unimodal distribution of habitable landscapes in terms of mean Late Pleistocene precipitation, with 30% of the refugial area receiving an average of 400–600 mm precipitation, 25% receiving 600–800 mm, with both 800–1000 and 1000–1200 mm precipitation present across 17% of the narrow refugia area. Within the broad refugia, a bimodal distribution is present for mean Late Pleistocene precipitation, with 15% of the area receiving 400–600 mm and 14% of the area receiving 1200–1400 mm. The two refugia observe substantial overlaps in the range of 400–1200 mm mean Late Pleistocene precipitation. However, a larger area within this range is covered by the broad refugia, accounting for increased tolerance of variability beyond the limits of the narrow refugia despite shared central tendencies. The most substantive difference between the two refugia is the proportion of more humid environments potentially available to human populations in the broad refugia, with 25% of the total area within this threshold receiving mean Late Pleistocene precipitation greater than 1403 mm. By contrast, only 3% of the area within the broad refugia receives less than 248 mm mean Late Pleistocene precipitation.

Considerable variability in the scale of precipitation is observed between the refugia identified here, such that landscapes considered habitable at one end of the spectrum could still be inhospitable to a population adapted to inhabiting landscapes at the other end of the spectrum. While some of this variability is clearly expressed spatially, there is also considerable variability in precipitation through time. Persistently habitable landscapes may be considered as those that not only fall between the precipitation CIs we identify, but also in which the amount of precipitation is more consistent through time. We examined patterns of absolute and proportional variability in precipitation within each refugia. Figure 1*b* illustrates the standard deviation (s.d.) of mean annual precipitation through the Late Pleistocene, expressed in mm. Two-thirds of the area within the 68% refugia experience an s.d. of 100 mm or less throughout the Late Pleistocene, indicative of low absolute variability in precipitation. A larger proportion of the area within the broad refugia experiences greater variability in precipitation, with over a quarter (26.7%) experiencing an s.d. of 100–150 mm, with a further 27% of this area seeing even more extreme variability. This extreme variability is largely constrained within the interior of West Africa and smaller pockets along the coast of Angola and Eritrea. Areas of notably low variability include the Maghreb, South Africa and southern Ethiopia/northern Kenya. Within the narrow refugia, extreme variability can be observed to impact the thin band of habitable landscapes at the southern Sahel margins. Figure 1*c* illustrates the coefficient of variation of mean annual precipitation, which expresses variation with respect to the mean accounting for the fact that, for example, a change of 100 mm precipitation may be significantly more pronounced in more arid than more humid regions. The majority of refugia areas see variation of up to 15% of the mean precipitation value for both the narrow refugia (69% of total refugia area) and broad refugia (66% of total refugia area). Eritrea is identified as a hotspot for high variability in precipitation through the Late Pleistocene, as is the Angolan coastline. A substantial contiguous area of high variability is observed spanning the southern Sahel zone. Here, the Congo and Lake Victoria Basins, as well as much of Ethiopia and northern Kenya present a large region of low proportional variability, along with the Maghreb and South Africa. Within the narrow refugia, the thin band of habitable landscapes across the Sahel is again identified as highly variable.

### (b) Biome changes within precipitation refugia

**Figure 2.**
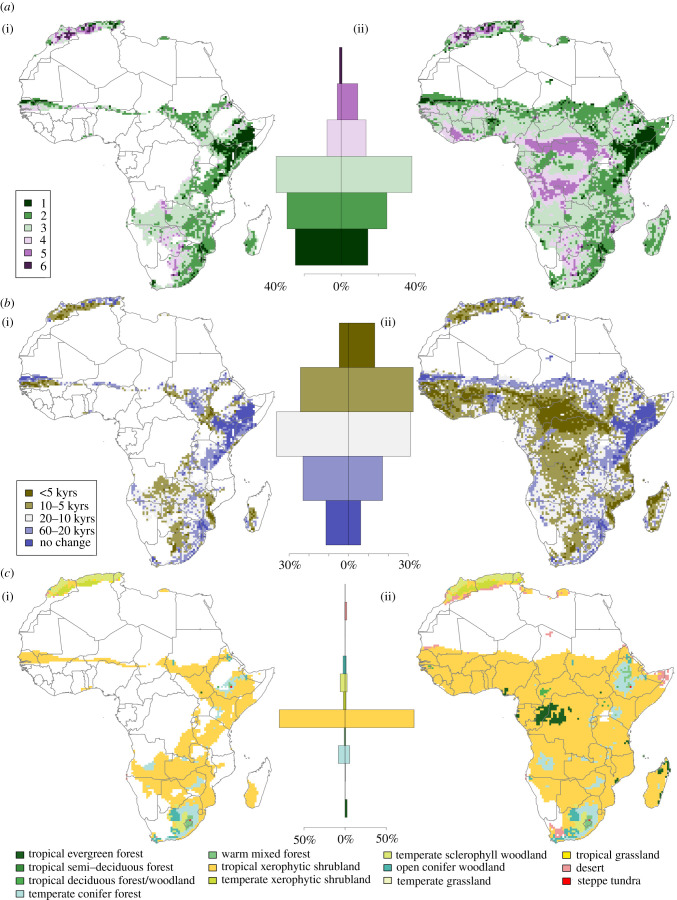
Maps of changing patterns of biome distributions based on narrow refugia (i) and broad refugia (ii); mean annual precipitation CIs illustrating (*a*) the count of alternate biomes present throughout the Late Pleistocene, (*b*) the frequency of alternation between biomes, and (*c*) the modal biome, with histograms (centre) displaying the percentage area of each refugia covered in each category of the respective maps within narrow (centre-left) and broad (centre-right) refugia.

We examined the modulation of biomes within the narrow and broad refugia throughout the Late Pleistocene. Whereas precipitation thresholds present a gross means to evaluate how environmental change may have modulated human habitability and persistent occupation of alternate landscapes, the changing distribution of biomes presents a rapid index to summarize a broader range of climatic impacts on localized ecologies. Long-term stability in the presence of a particular biome in any given location may facilitate the predictability of resource landscapes and thus promote their suitability for enduring occupations by human populations. We evaluated the number of unique biomes found per grid cell across Africa throughout the Late Pleistocene to identify locations in which there has been stability in biome distribution and those locations which have seen modulation between multiple alternate biome states (figure 2*a*). The largest proportions of both the narrow and broad refugia have seen three alternate biome types present at some point across the Late Pleistocene, accounting for 39% of each refugia area. Similarly, large proportions of both precipitation refugia have seen two alternate biome states (narrow refugia: 34%; broad refugia: 28%). The narrow refugia has the largest proportion of its area with a stable biome presence throughout the Late Pleistocene (11%) as opposed to the broad refugia (6%), with the latter showing higher proportional area with 4–6 biome states present.

Late Pleistocene biome stability is most concentrated in southeast Ethiopia, Somalia, north and east Kenya. Elsewhere, only a single biome state throughout the Late Pleistocene is present in isolated pockets in southern Mozambique, the Dahomey Gap, the Senegal Valley, Upper Niger Valley and in the Maghreb. A latitudinal band across the southern Sahel marks a region that sees alternation between two biome states through the Late Pleistocene, whereas a comparable, broadly longitudinal band can be identified running parallel to the eastern African coastline between northern Tanzania and southern Mozambique. The highest numbers of biome states are identified in the Maghreb, with more substantial contiguous areas showing between four and six alternate biomes present throughout the Late Pleistocene in the Congo and Lake Victoria basins, the West African coast between Ghana and Liberia. We next examined the frequency of changes in biome states between consecutive time slices (figure 2*b*). Ten per cent of the narrow refugia and 6% of the broad refugia observe no changes in biome state throughout the Late Pleistocene. Low-frequency changes in biome state are typically seen adjacent to those areas in which no change is seen, such as across the southern Sahel Belt and Dahomey Gap, extending through the southern Rift Valley in Tanzania, or surrounding the junction of Mozambique, Zimbabwe and South Africa. Much of the Congo and Lake Victoria basins and West Africa show large contiguous areas of particularly frequent changes in biome state. Much of the Maghreb similarly sees frequent biome changes, alongside notable pockets along the eastern African coastline and southern African interior. Infrequent changes in biome states, occurring every 60–20 kyrs, occur across 23% of the narrow refugia, but a more reduced proportion of the broad refugia, accounting for 17% of its total area. Within both refugia, large areas (narrow refugia: 37%; broad refugia: 33%) saw alternation of biome states at a frequency of 20–10 kyrs. Changes in biome occur every 10–5 kyrs across 24% of the narrow refugia, with 5% of the total refugia area seeing changes more frequently than every 5 kyrs. These high-frequency changes in biome state are more commonplace with the broad refugia, with 33% of the refugia area experiencing changes every 10–5 kyrs, and a further 13% experiencing more frequent changes. We note here that patterns of biome stability or low-frequency change do not necessarily correlate with low variability in annual precipitation, with substantial variability in precipitation possible within a given biome, as well as minor variability in precipitation (as well as other climatic factors) potentially sufficient for repeated alternations across a key threshold for two biome states.

A total of 16 alternate major biomes are found to exist within Africa during the Late Pleistocene (figure 2*c*). We identified the most frequent biome type occurring within each refugia during the Late Pleistocene, calculated as the modal biome type within each cell over time, to present a simple characterization of the ecology of precipitation refugia that we identify. This overwhelmingly identifies tropical xerophytic shrubland as the dominant biome type within refugia, spanning 84% of the broad refugia and 80% of the narrow refugia. Within the narrow refugia, temperate conifer forest (8%), temperate sclerophyll woodland (6%), open conifer woodland (2%) and temperate xerophytic shrubland (2%) cover substantive areas, with all other biome states present as the modal type in less than 1% of the refugia area. The Maghreb is typically composed of temperate woodland and shrublands, with the interior of southern Africa composed of a mosaic of sclerophyll and conifer wood and forest. Within the broad refugia, temperate conifer forest (5%) covers a substantive area of the refugia, with sparser distributions of tropical evergreen forest and temperate sclerophyll shrubland (both 3%), desert (2%) and open conifer woodland and temperate xerophytic shrubland (both 1%) covering substantive areas, with other biome states present as the modal type in less than 1% of the refugia area. Tropical forests are concentrated in the central Congo Basin, with temperate conifer forests showing a more extensive distribution from southern Africa to include presence in the Ethiopian Rift and eastern Lake Victoria Basin, along with a patchy distribution elsewhere across southern Central Africa.

The prominence of tropical xerophytic shrubland is supported when the datasets spanning the Late Pleistocene are pooled together to explore the gross frequency of alternate biome types. Tropical xerophytic shrubland spans 71% of all cells of the narrow refugia and 69% of the broad refugia. Within the narrow refugia, temperate conifer forest spans 11% of the pooled dataset, with temperate sclerophyll woodland (5%), tropical deciduous forest/woodland (4%) and open conifer woodland (3%) indicating a substantial persistent presence. Temperate conifer forest (7%) plays a more limited role within the broad refugia, alongside tropical deciduous forest/woodland (6%), tropical evergreen forest (4%), desert, temperate sclerophyll woodland, and tropical semi-deciduous forest (all 3%), and tropical savannah (2%).

### (c) Open and forested landscapes within precipitation refugia

**Figure 3.**
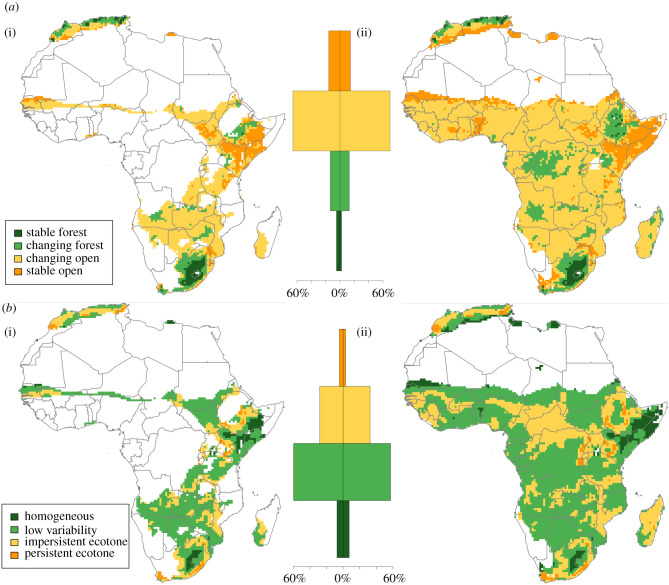
Maps of narrow refugia (i) and broad refugia (ii); illustrating (*a*) distribution of stable and changing open and forest biomes, and (*b*) the distribution of ecotonal habitats, highlighting the limited presence of persistent open:forest ecotones. Histograms (centre) display the percentage area of each refugia covered within each category displayed on respective maps within narrow (centre-left) and broad (centre-right) refugia.

Tropical xerophytic shrubland is clearly the dominant individual biome within Africa over the Late Pleistocene, with a range of woodland/forest biomes also constituting smaller but substantial components within both refugia. To contrast the distribution of permanence of open and forest landscapes, we simplified biome models for the Late Pleistocene; though recognizing that this overlooks the substantive differences in resource structures that various forest/woodland biomes support, such as between temperate tropical forests and tropical evergreen forests, this was undertaken to explicitly highlight the potential of boundaries between forest and open biomes as critical ecotones, highlighted by the foregoing review as potentially significant for human populations. We categorized biomes as either open or forests and established whether or not a change between these two states occurred during the Late Pleistocene, resulting in a bivariate classification of stable or changing and open or forest (figure 3*a*). Within both precipitation refugia, changing open landscapes comprise approximately two-thirds of the refugia areas (narrow refugia: 66%; broad refugia: 70%), with stable open landscapes comprising similar proportions of the narrow refugia (16%) and the broad refugia (15%). Changing forests comprise 14% of the narrow refugia and 13% of the broad refugia, whereas a greater proportion of the narrow refugia sees stable forests (5%) in contrast with the broad refugia (2%).

Stable forests are concentrated in the interior of South Africa/Lesotho and the Maghreb coast, both of which are also associated with changing forests. Little persistent forest is observed in the Ethiopian Rift, and stable forests appear absent in the Congo Basin, though both regions preserve large areas of changing forests, along with the Lake Victoria and Zambezi basins. Stable open landscapes are concentrated in the Horn of Africa and southern Sahel, as well as the southern margins of the Maghreb. Much of west, central and southern eastern Africa constitute changing open landscapes. South Africa and the Maghreb document changes from stable forest to stable open landscapes within a relatively limited distance, as does the Ethiopian Rift to a lesser extent.

A substantial proportion of both refugia constitute landscapes in which there is flux between open and forested landscapes, requiring further examination of whether geographically stable persistent ecotones indeed existed during the Late Pleistocene. For each cell as the centre of a 9-cell neighbourhood, we calculated whether either or both open and forested landscapes were present across each time slice and the average of whether either one or both landscapes were accessible to each cell throughout the Late Pleistocene (figure 3*b*). Geographically stable ecotones between open and forest landscapes that persist throughout the Late Pleistocene are rare, with isolated patches in the Maghreb, the margins of the Ethiopian Highlands and Lake Victoria Basin, and along the South African coast, featuring in both refugia models. Ecotonal settings that are present for at least half of the Late Pleistocene surround and connect isolated elements of persistent refugia in each of these locations, with notable expansions in the northern Ethiopian Highlands and interior of South Africa. In addition, impersistent ecotones that are present for at least half the Late Pleistocene are more widely distributed in the Zambezi and Lake Malawi basins, and the Ivory Coast to Guinea. All of these to some extent feature in the narrow refugia and are more widely evident alongside impersistent refugia in the northern margins of the Congo Basin and Cameroonian Highlands within the broad refugia. Significant flux in the distribution of open and forested landscapes is observed over the Late Pleistocene, suggesting for much of the continent, ecotones were typically not geographically stable, but such stability does occur, and is present in multiple discrete locations in both refugia.

### (d) A case study of Late Pleistocene human occupation of eastern African refugia

**Figure 4.**
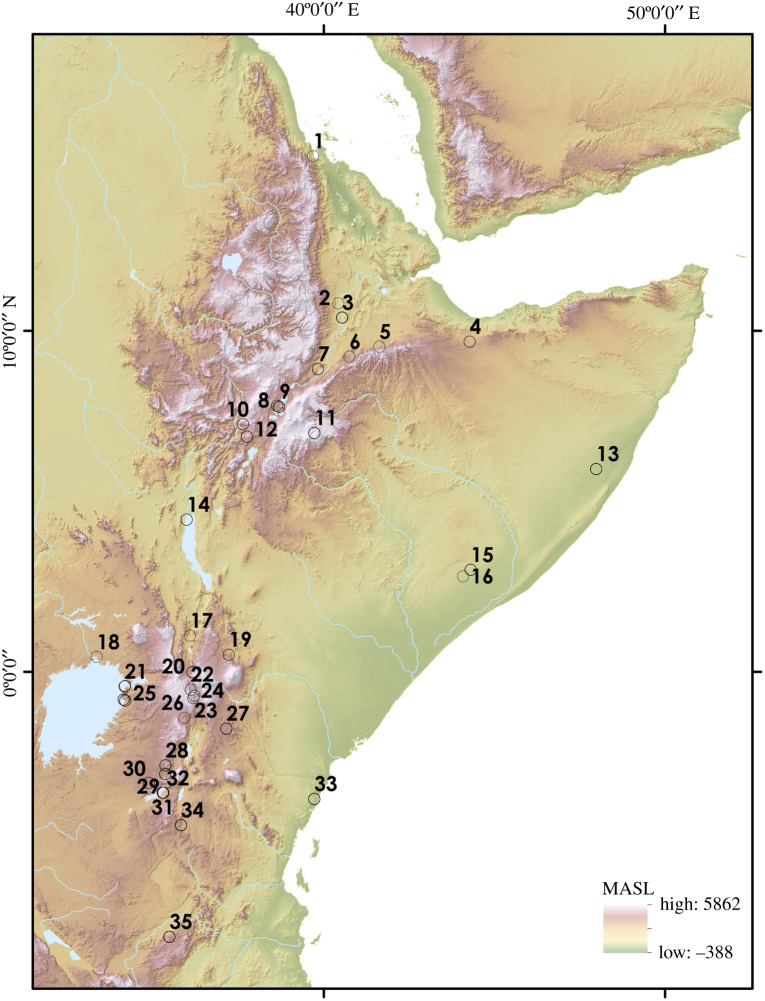
Map illustrating the distribution of sites with dated Late Pleistocene occupations in eastern Africa (data: SRTM (Source: NASA)): 1, Abdur; 2, Halibee; 3, Aduma; 4, Laas Geel; 5, Goda Buticha; 6, Aladi Springs; 7, FeJx2 and FeJx4; 8, Macho Hill; 9, Ziway-Shala; 10, Sodicho; 11, Fincha Habera; 12, Mochena Borago; 13, Mirsale Wells; 14, Omo; 15, Guli Waabayo; 16, Rifle Range Site; 17, Kapedo Tuffs; 18, Munyama Cave; 19, Shurmai; 20, Enkapune ya Muto; 21, Rusinga; 22, Nderit Drift; 23, Ol Tepesi; 24, Marmonet Drift; 25, Karungu; 26, Ntumot; 27, Lukenya Hill; 28, Nasera; 29, Victoria Cabera; 30, Naisiusiu; 31, Mumba; 32, Eyasi Shore; 33, Panga ya Saidi; 34, Kisese II; 35, Magubike.

Eastern Africa presents an ideal laboratory in which to explore the role of refugia in Africa during the Late Pleistocene, given the relatively large number of spatially diverse sites and dated occupation horizons, as well as ecological diversity within the refugial zones we identify. A total of 164 discrete occupations across 35 sites are identified in eastern Africa with a central age estimate within the Late Pleistocene (figure 4; electronic supplementary material, table S1), of which 126 (79%) are located within the narrow refugia we identify. Thirty (18%) occupations occur at sites beyond the limits of the narrow refugia, but within the limits of the broad refugia. Five occupations across three sites occur beyond the boundaries of the broad refugia we identify. These distributions support our conservative use of ethnographically derived data in order to identify past refugia and provide a baseline against which to further constrain potential past refugia based on palaeoenvironmental proxy datasets from excavated occupation horizons. To explore patterns of occupational continuity and ecological diversity, we divide the eastern African Late Pleistocene archaeological record in two for further analysis, distinguishing between northern sites, located within the Horn of Africa and the Ethiopian Rift, and southern sites, located within the Kenyan and Tanzanian Rift, the Lake Victoria Basin, and the Kenyan coast.

**Figure 5.**
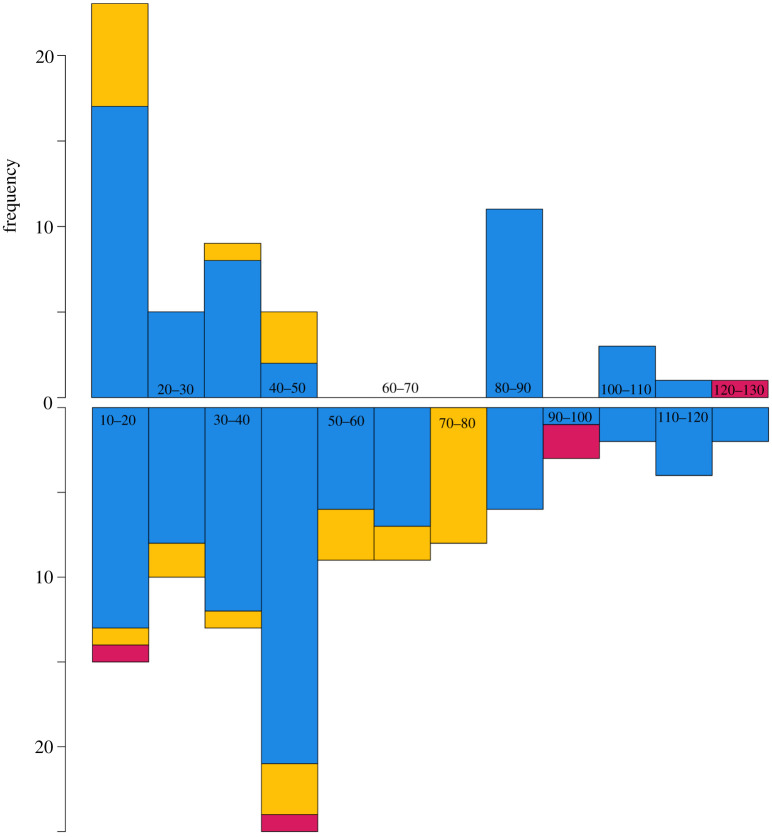
Histograms illustrating frequency of occupation of Late Pleistocene sites in eastern Africa within the narrow refugia (blue), broad refugia (yellow) and beyond either refugia (red) based on central age estimates of dated stone tool assemblages within 10-thousand-year bins between 10 and 130 ka, indicating impersistent occupation of northern sites (top) until 40–50 ka onwards, whereas occupation of southern sites (bottom) has been continuous throughout the Late Pleistocene.

**Figure 6.**
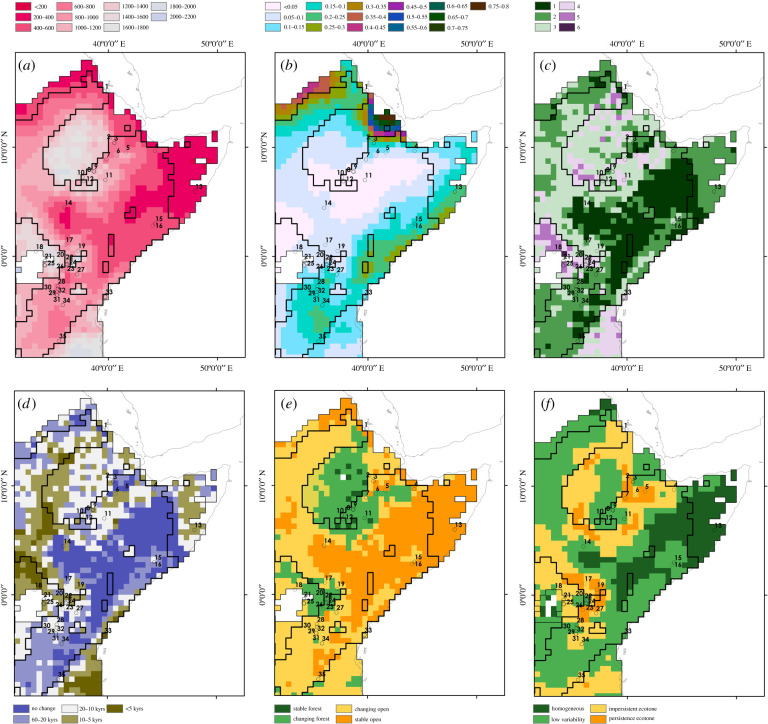
The distribution of Late Pleistocene sites in eastern Africa (figure 4 for site labels) with respect to the narrow refugia (thick black line) and broad refugia (thin black line), with regions beyond either refugia shown as blank, illustrating mean annual precipitation (*a*), coefficient of variation for precipitation (*b*), number of alternate biomes present throughout the Late Pleistocene (*c*), the frequency of alternation of biomes (*d*), characterization of stable or changing open or woodland landscapes (*e*), and the distribution of ecotones between open and woodland landscapes (*f*).

We identify 58 occupation horizons in the northern sites that occur during the Late Pleistocene (1–15 on figure 4), 47 of which occur within the narrow refugia, with a further 10 falling within the broad refugia, and one beyond either refugia. Occupation of the northern sites is impersistent throughout the Late Pleistocene (figure 5, top). A single occupation at Abdur Reef, occurring beyond the refugia we identify, occurs at the start of MIS 5. Occupation of the narrow refugia is first seen at Halibee (126–95 ka), Omo (111–104 ka) and Herto (100–80 ka), with a substantial gap before the next phase of inhabitation of the northern sites, commencing within MIS 3. Notably, occupation of Mochena Borago (50–37 ka), located within the boundary of the broad refugia, probably commences prior to further inhabitation of the narrow refugia. The chronology for the earliest occupations of Goda Buticha, located within the narrow refugia, is currently poorly constrained (70–22 ka), with a firmer chronology for occupation of this zone apparent for occupations beginning at Laas Geel (43–41 ka) and Fincha Habera (43–33 ka), and broadly continuous to the end of the Late Pleistocene. Inhabitation of Mirsale Wells (16–15 ka) presents a further, recent occupation within the limits of the broad refugia. Occupations of the Ethiopian Rift in the Late Pleistocene (sites 3–12 on figure 4) are found in landscapes which typically see the presence of three to four biomes, alternating on 5–10 thousand year timescales, and characterized as changing woodlands (figure 6). A group of sites (sites 2–7) are found to occupy persistent or impersistent ecotones, whereas the remaining Ethiopian Rift sites occupy landscapes with lower habitat variability. By contrast, other sites in the Horn of Africa occupy more stable, open and homogeneous Late Pleistocene landscapes.

Of 106 occupation horizons found within the southern sites during the Late Pleistocene, we identify 82 that are located within the narrow refugia, a further 20 within the broad refugia and four occurring beyond the refugia boundaries we identify. Occupation of the narrow refugia is broadly continuous throughout the Late Pleistocene (figure 5, bottom), with error ranges of age estimates for occupations overlapping for all but one thousand years, between inhabitation of Victoria Cabera 2 (92–70 ka) and Mumba Upper VI A (69–58 ka). Additional continuous occupation beyond the narrow refugia but within the broad refugia limits appears at occupations of Panga Ya Saidi on the Kenya coast, which are broadly continuous from 86–68 ka and continue into the Holocene. A number of sites in the Lake Victoria Basin span the boundary of the broad refugia, with occupations at Karungu within the broad refugia (116–42 ka) and beyond at Rusinga (128–42 ka) occurring between MIS 5–3, though without evidence for any clear continuity. A further late occupation beyond the broad refugia in the Lake Victoria Basin is seen at Munyama Cave approximately 18 ka. Southern sites located within the narrow refugia typically occur in landscapes in which two to three alternate biome types occur during the Late Pleistocene, with changes occurring at 10–20 thousand year timescales (figure 6). By contrast, occupations within the broad refugia and beyond are associated with landscapes in which a larger number (four to five) of alternate biomes occur during the Late Pleistocene, with higher frequency changes including up to every five thousand years at Panga ya Saidi. Sites at higher elevations concentrated in southern Kenya are typically associated with changing woodland habitats and include occupations of persistent ecotones, rather than changing grassland habitats in which Late Pleistocene occupations in the Victoria Basin, Tanzanian Rift and Kenyan coast are situated, where impersistent ecotones or low-variability habitats occur.

## 4. Discussion

The potential refugia we identify suggest persistent occupations of between 27.5% and 66.3% of Africa throughout the Late Pleistocene were possible. In both narrow and broad refugia, potential zones of habitability are geographically diverse rather than centring on any single region. These results, in part, support the breadth of landscapes previously proposed as potential refugia for Late Pleistocene human populations. Eastern Africa has historically played a key role in human refugia models for Africa, in part owing to research history and the distribution of fossiliferous deposits. Nevertheless, our models, and particularly the narrow refugia model, supports this region as providing continuity in terms of suitable precipitation regimes, consistency of biome distributions and the presence of persistent open/woodland ecotones. Our evaluation of the Late Pleistocene archaeological record of eastern Africa corroborates the potential for this region to have provided an important refugia for human populations. This is particularly apparent from the continuous occupation of Kenyan and Tanzanian Rift throughout the Late Pleistocene. Beyond this, our models also highlight the significance of the Maghreb and southern African coastline as persistent refugia and, in each case, compressed environmental variability has been highlighted, suggesting that a diverse range of habitable landscapes were accessible within limited geographical ranges. Our models draw attention to two other refugial regions that are more broadly overlooked: the Zambezi Basin and the southern margins of the Sahel Belt. The former shows a large, contiguous habitable area whereas the latter exhibits a more sinuous distribution but identifies potentially stable refugia in the Senegal Basin, with limited variability for both precipitation and biome state. The most notable difference between the narrow and broad refugia models relates to the habitability of the Congo Basin and West Africa, which frequently sees high levels of humidity significantly beyond the upper threshold of the narrow refugia.

Periods of connectivity and isolation between habitable areas has been suggested to have been a key driver of hominin population diversity [18]. Broad geographical connectivity can be identified between refugial zones in eastern Africa, the Zambezi Basin and southern Africa within the narrow refugia. The Senegal Basin refugia is not persistently connected to eastern African refugia, with high levels of absolute and relative variability in precipitation within the narrow refugia across the southern margins of the central Sahel Belt probably limiting east-west connectivity. By contrast, north African refugia are isolated from one another and from sub-Saharan refugia, implying that connectivity with other parts of the continent was not persistent and probably reliant on pulses of heightened humidity [61]. Such patterns of fragmentation and coalescence present typical predictions from a refugia model that can be tested against archaeological, fossil and genetic evidence as contributing to the generation of population structure [5,6]. However, sub-Saharan refugia are largely contiguous within the broad refugia model, prohibiting straightforward predictions for patterns of human phylogeography. In this case, there is greater potential for adaptation to divergent precipitation regimes, providing an alternative mechanism for divergence between populations, such as between groups occupying landscapes with 400–600 mm and 1200–1400 mm mean annual precipitation. As such, the persistent habitability of large regions of Africa within the broad refugia may not have necessarily resulted in large-scale population connectivity, presenting an alternate hypothesis to refugia models in which disconnected populations are simply adapted to different local conditions.

Adaptation to a single biome necessitates biome stability to identify refugia and would have resulted in highly restricted population distributions. Yet the majority of both precipitation refugia experience some extent of alternation between biome types that is also characteristic of changes between open and forest landscapes. Our review of previous studies highlights the potential importance of being able to access alternate biomes to the definition of human refugia, and therefore tolerance or adaptation to some flux in ecology would enable occupation of significantly larger areas within our identified refugia. Notably, in our evaluation of Late Pleistocene occupations of eastern Africa, we identified that occupations within homogeneous and stable biomes were rare, with the majority of occupations associated with more variable habitats. Here, the chronology, frequency and geography of biome flux is likely to be significant with regard to the emphasis placed on behavioural adaptation or mobility strategies for past human populations to manage such changes. Spatial and temporal variability are frequently suggested to elicit distinct evolutionary responses; many theoretical models associate the former with the emergence of generalism and the latter with the evolution of behavioural or phenotypic plasticity [62,63]. Plasticity has been argued to have been vital for Late Pleistocene dispersals within and beyond Africa, probably buffering populations against rapid habitat disturbance during periods of increased environmental fluctuation while also encouraging the development of new adaptations which helped to facilitate the occupation of new landscapes during periods of less perturbation [64,65]. Variability selection, as originally coined by Potts [66] to describe the evolution of plasticity in the face of climatic variability, is one of the major hypotheses regarding the emergence of *Homo sapiens* and was borne from observations that MSA innovations are first seen in eastern Africa during periods of dramatic environmental perturbation. Addressing such issues is becoming increasingly pertinent to research in evolutionary anthropology, but is difficult via the use of single or even multiple geographically specific core records (e.g. [2]). The modelled datasets employed here allow us to characterize both the geographical and chronological extent of refugia as well as their internal variability. The latter may be particularly important in future studies of Late Pleistocene hominin adaptive and dispersal patterns, especially in light of our analysis of the eastern African record which found that many sites were located in areas of strong habitat perturbation.

Tropical xerophytic shrubland is clearly identified as the most abundant biome within both refugia, both in terms of the most common biome present in any given location and in terms of gross abundance throughout the Late Pleistocene. This in part probably reflects its widespread distribution in Africa throughout the Late Pleistocene, and precipitation envelope used in this analysis, which is in part corroborated by our examination of the eastern African record. Given its abundance, we may anticipate human adaptive strategies to have focused on engaging with subsistence resources associated with this biome, with mobility providing a ready strategy to ensure continued accessibility of these resources. A growing body of research is illuminating the fact that large areas of Africa may possess suitable climates for forest cover; however, natural and anthropic disturbance can prohibit the establishment of forested biomes, resulting in more extensive coverage by savannah-type landscapes [67–69]. The presence of other biomes within refugia is spatially distinct and more circumscribed, with the Maghreb, Ethiopian Highlands, Congo and Lake Victoria basins and interior of South Africa preserving alternate forest habitats. The appearance of forests within these regions plays an important role in the identification of major ecotones and represents contexts in which distinctly composed ecotonal habitats provide a discrete buffer against either the spread of forest or its destruction through alternate means of disturbance [68]. This distribution is notably shared with many classic sub-Saharan refugia for African birds and mammals [20,21], with impersistent ecotones also identified in Upper Guinea, the Cameroon Highlands and the southern African interior. The extent and role of human adaptation to Late Pleistocene forests within Africa has typically been overlooked and, as a result, it remains unclear whether ecotones between open and forested habitats may have been critical features of human refugia owing to direct exploitation of forest resources or because they comprise key refugial zones for subsistence resources typically found beyond the forest, or a combination of the two. Nevertheless, our evaluation of the eastern African record suggests that the widespread stable, homogeneous open landscapes of the region do not preserve extensive or continuous records of Late Pleistocene inhabitation, and rather that human occupations are more typically found where ecotones between open and forested habitats occur.

Stone Age occupations are broadly distributed across the narrow refugia within Africa throughout much of the Late Pleistocene. Northwest and South Africa are notable for sharing patterns of spatially compressed precipitation and ecological variability, as well as their coastal location. Elsewhere, only the coastlines of Kenya and southern Somalia in the east and Senegal/Gambia in the west fall within the narrow refugia. MSA occupations are prevalent within landscapes that fall below precipitation thresholds of either refugia model, such as the Sahara, but are restricted to phases of enhanced humidity, notably MIS 5 [70]. The Lesotho Highlands are a consistent feature of both refugia, whereas the Ethiopian Highlands only feature within the 95% refugia. The earliest currently known occupation in Lesotho dates to 80 ka [38], whereas the history of highland occupation in Ethiopia is shorter, dating to 40 ka [36]. This potentially indicates that high-altitude regions demanded specific adaptations and, given that their occupation has not spanned the entire Late Pleistocene, may be excluded from more general models of African refugia for human populations. A number of MSA sites, now dating to the start of the Late Pleistocene, are found in the Senegal Basin [71] and upper reaches of the Niger Valley [72], both of which coincide with our refugia models. Yet considerable debate surrounds the chronology and ecological context of wider occupation of west and central Africa for much of the Late Pleistocene [45]. Reliably dated evidence for occupation of these regions beyond the limits of the narrow refugia (but within those of the broad refugia) are restricted to the past 30 ka [73]. It remains problematic to evaluate whether more widespread but undated evidence for occupation of these regions were the result of distinct adaptations to the forested landscapes present today, or whether occupations were more temporary and related to punctuated phases of increased aridity, creating more open habitats.

Through the persistent presence of a given habitat, refugia should support enduring adaptations. Among the refugia zones we identify, it is the Senegal Basin that exhibits the greatest stability, falling within the middle of the precipitation range of the narrow refugia, with low absolute and relative flux in precipitation, with limited diversity and alternation of biome states through the Late Pleistocene. Here, MSA occupations are known throughout the Late Pleistocene [71,74,75], with the recent discoveries of sites spanning the Late Pleistocene-Holocene transition post-dating major technological changes seen across much of the continent by 20–50 kyrs [47,76]. Long-term palaeoclimatic and biome stability in this region may therefore be factors contributing to explanations of the persistence of MSA technologies in this region. Our analyses of the eastern African Late Pleistocene archaeological record highlight persistent occupations of the narrow refugia within the southern Rift Valley, with discontinuous occupation of the narrow refugia in the northern part of this region, and sporadic occupations in the broad refugia and beyond. Occupations at Panga ya Saidi, coastal Kenya [46], indicate a persistent presence within the broad refugia from *ca* 80 ka onwards, marking a departure from the overall regional pattern. As this site is associated with the earliest appearance of Later Stone Age technologies both in the region and the continent, this may suggest a role for behavioural adaptation enabling expansions into new palaeoclimatic and habitat settings. Further study of variability within refugia may be critical to exploring how populations successfully adapted to unfamiliar habitats and expanded into new landscapes.

Our evaluation of Late Pleistocene refugia for human populations in Africa, like those that we succeed, are necessarily limited by the datasets available and how they are employed. The extent to which our use of ethnographic datasets, constrained by standard CIs, may reflect patterns of Late Pleistocene population adaptation can be tested against archaeological records. Examining the palaeoclimatic context and distribution of Late Pleistocene archaeological sites provides a direct means to refine such parameters without extrapolating from ethnographic data [77]. However, this may only be possible for those regions with the most extensive and well-dated archaeological records and thus is shaped in part by research history. In practice, comparisons of ethnographic and archaeological data sources may offer useful predictions for the distribution of Late Pleistocene refugia, which may include regions in which archaeological records remain poorly resolved. Similarly, our use of a single model dataset that spans Africa at 1 kyr resolution enables a continent-wide assessment of potential refugia distribution throughout the Late Pleistocene. Further refinement of both the spatial extent of these refugia, as well as how they relate to alternate biome structures and ecotones, can be achieved at regional scales with reference to suitable proxy records.

As areas of persistently habitable landscape, refugia are of particular importance to the population dynamics of any species; they govern not only patterns of geographical extent through time but also the differences and similarities between sub-populations that may emerge during periods of isolation and coalescence, respectively. Such differences and similarities are likely to have been amplified in a highly cultural species such as *Homo sapiens*; the unique long-term record of behavioural change provided by the archaeological record allows us to study relationships between regional populations, but this can only be done against the backdrop of a comprehensive, spatially explicit, quantitative description of likely refugial zones. The analyses above provide a first step towards this description and should provide a stimulus to the future development of regionally focussed examinations. Our use of high-resolution climatic models at a continental scale has enabled us to distinguish between those refugial zones offering relatively stable, consistent environments and those providing substantial variability within the broad envelope of habitable conditions; this previously unattainable result merits further examination, particularly in relation to the patterns of adaptation that these contrasting conditions may have produced. Analyses of the archaeological trajectories in these contrasting types of refugia should provide substantial insight into how our ancestors came to terms with the challenges of both spatial heterogeneity and temporal fluctuations in the availability of their favoured habitats.

## Data Availability

The data are available from the Dryad Digital Repository: https://doi.org/10.5061/dryad.sbcc2fr84 [78].
